# Peer review in the age of machines: Who evaluates science now?

**DOI:** 10.5114/bta/220048

**Published:** 2026-03-31

**Authors:** Maciej Kukula

**Affiliations:** Discovery, Science, Transformation & Growth, DSTG, Bedford, NH, USA

Artificial intelligence (AI) systems are evolving from assisting research to evaluating it. This shift is occurring rapidly, albeit with limited consensus on monitoring standards. In a 2025 survey of more than 5,200 scientists conducted by *Nature*, over 90% of respondents agreed that using generative AI to edit or translate their own research paper is acceptable (Kwon 2025). This widespread uptake reflects a broader trend: researchers who integrate AI into their workflow tend to perform more efficiently, with measurable gains in their productivity. A 2024 preprint article reports that scientists who used AI published 3.02 times more papers, received 4.84 times more citations, and became research project leaders 1.37 years earlier than those who do not use AI (Hao et al. 2026). This rapid acceleration raises broader questions about how deeply AI should be embedded in scientific practice and what forms of oversight are needed to ensure that efficiency gains do not come at the expense of scientific rigor.

The trend of using AI in peer review is also gaining momentum. Recent evidence indicates that large language models (LLMs) acting as peer reviewers recommend acceptance of entirely AI-written manuscripts in most cases (82%), even when methodological weaknesses are detectable (Chawla 2025). A survey of more than 1,600 researchers across 111 countries found that more than 50% of them used AI tools when reviewing their manuscripts (Naddaf 2026). Many researchers employ AI to write review reports, summarize manuscripts, or identify gaps and references. These practices frequently come into conflict with publisher policies that restrict uploading unpublished work to external systems. The result is a growing divergence between formal guidance and routine behavior. The survey report calls on publishers to respond to the increasing use of AI in scientific publishing and implement policies better suited to the “new reality.” In response, *Frontiers* has launched its own in-house AI platform for peer reviewers across all its journals (Naddaf 2026).

These developments prompt important questions: Where should the boundary lie between human judgment and automated assistance? To what extent can AI meaningfully evaluate scientific novelty or methodological soundness? A recent *Nature* report adds further nuance to this shift, describing a multi model AI system designed to help peer reviewers produce clearer, more constructive, and more polite feedback. Early evidence shows that reviewers who receive AI generated suggestions tend to revise their reports and provide more specific comments, although the study found no measurable improvement in acceptance decisions or in the scientific quality of revised papers. These findings highlight both the potential and the current limitations of AI mediated peer review assistance (Zhao 2025). Addressing these questions may require empirical studies comparing AI assisted and human only evaluations, as well as frameworks that clarify which aspects of peer review can be delegated to AI without compromising accountability.

New publishing infrastructures are emerging to institutionalize AI participation. The preprint platform aiXiv accepts AI authored and AI reviewed work and guides authors through revisions based on chatbots’ feedback (Jones 2026). aiXiv’s infrastructure can support thousands of submissions, and it generates reviews within a few minutes, in contrast to the several months or years required for conventional peer review. Proponents frame this model as a response to reviewer overload; however, critics warn that increasingly persuasive machine generated manuscripts may pass through automated filters with insufficient scrutiny.

As these systems scale, the scientific community must consider how the future of research will look when both authorship and evaluation can be partially or fully automated. Will AI become a routine co participant in knowledge production, remain primarily supportive, or ultimately replace human evaluators altogether? In parallel with these developments, there are broader debates regarding regulations for AI. Even though recent policy changes are often described as “deregulation”, new analyses show that governance isn’t disappearing – it’s being reorganized. Executive power, industrial policy, funding decisions, and federal overrides of state authority are reshaping the conditions for conducting AI based research and evaluation. Instead of vanishing, the oversight process is taking a new form. Recent developments in the United States further illustrate this shift. Following the December 2025 announcement of the “One Rule” executive order – which framed federal preemption of state level AI laws as a move toward deregulation – the governance landscape has been reshaped through mechanisms such as executive discretion, industrial policy, ownership stakes in private firms, immigration controls, and the redirection of research funding. Although presented as reducing regulatory burden, these actions represent a more centralized and less transparent form of oversight, demonstrating that the absence of formal rulemaking does not equate to the absence of governance (Nelson 2026).

Taken together, these trends show how AI systems increasingly influence both the production and assessment of scientific work, while governance mechanisms operate earlier in the research pipeline. These shifts also raise the issue of authorship and responsibility: where does the scientist’s contribution end and the AI’s contribution begin, and how should this boundary be defined in practice? Several countries, including the United States, have begun developing regulatory frameworks – such as federal AI guidelines and risk management standards – that may shape how AI can be used in scientific evaluation. Clear legal definitions and transparent disclosure requirements will be essential to ensure responsible use. The central challenge in this new order is not simply whether AI should participate in evaluating science, but how credibility can be maintained when knowledge creation and assessment become intertwined with technological and political forces. Clear disclosure standards, independent verification, and transparent governance are the critical factors for accelerated publication models to sustain scientific trust (Nelson 2026).

## References

[cit0001] Chawla DS. 2025. AI peer reviewers are fine with AI-fabricated papers. Chem Eng News 11. https://cen.acs.org/research-integrity/AI-peer-reviewers-fine-AI/103/web/2025/11.

[cit0002] Hao Q, Xu F, Li Y, Evans J. 2026. Artificial Intelligence Tools Expand Scientists’ Impact but Contract Science’s Focus. Nature 649(8099): 1237–1243. 10.1038/s41586-025-09922-y. 41535462

[cit0003] Jones N. 2026. This AI can improve your peer review – and make it more polite. Nature 651: 15–16. 10.1038/d41586-026-00536-6. 41731081

[cit0004] Kwon D. 2025. Is it OK for AI to write science papers? Nature survey shows researchers are split. Nature 641: 574–578. 10.1038/d41586-025-01463-8. 40369145

[cit0005] Naddaf M. 2026. More than half of researchers now use AI for peer review – often against guidance. Nature 649: 273–274. 10.1038/d41586-025-04066-5. 41398388

[cit0006] Nelson A. 2026. The mirage of AI deregulation. Science 391(6782): eaee4900. 10.1126/science.aee-4900. 41538433

[cit0007] Zhao C. 2025. A new preprint server welcomes papers written and reviewed by AI. Science 390(6779): 1202–1203. 10.1126/science.aee8377. 41411434

